# EXAMINING THE NURSING HOME INNER SETTING CHARACTERISTICS THAT LEAD TO THE SUCCESSFUL IMPLEMENTATION OF PAL CARDS

**DOI:** 10.1093/geroni/igac059.441

**Published:** 2022-12-20

**Authors:** Kamryn Kasler, Megan Kelley, Miranda Kunkel, Reese Moore, Faith DeVengencie, Alexandra Heppner, Kimberly Van Haitsma, Katherine Abbott

**Affiliations:** Miami University, Oxford, Ohio, United States; Miami University, Oxford, Ohio, United States; Miami University, Oxford, Ohio, United States; Miami University, Oxford, Ohio, United States; Miami University, Oxford, Ohio, United States; Miami University, Oxford, Ohio, United States; Pennsylvania State University, University Park, Pennsylvania, United States; Miami University, Oxford, Ohio, United States

## Abstract

Implementation efforts of evidence-based practices focusing on the nursing home setting remain understudied. The purpose of this study was to explore the role of the Consolidated Framework for Implementation Research (CFIR) inner setting domain on the implementation of PAL Cards. Monthly qualitative interviews (n=50) with project champions (n=16) were audio-recorded, transcribed, checked for accuracy, and coded using the domain “Inner Setting” from the CFIR in Dedoose. Major themes emerging from the data included networks and communication (e.g., including PAL Card information in employee newsletter) among staff, compatibility of the PAL Cards (e.g., aligns with community values and fits into existing workflows), and available resources (e.g., adequate time and staff to implement the QIP). High-quality communication channels to educate and collaborate were shown to be an integral component of successful implementation. Implications for policy and practice will be discussed.

